# Exploring Growth Pattern and Candidate Genes for Chicken Spur

**DOI:** 10.3390/ani16111577

**Published:** 2026-05-22

**Authors:** Hong Yuan, Qianhui Liao, Zhuliang Yang, Zhen Zhang, Jianneng Li, Fuqiu Li, Yunsheng Wang, Biyan Zhou, Jintang Luo, Xiurong Yang

**Affiliations:** 1 College of Animal Science and Technology, Guangxi University, Nanning 530004, China; yuanhong19970610@163.com (H.Y.); chanll05@outlook.com (Q.L.); yangzl@st.gxu.edu.cn (Z.Y.); zhangz18453709401@163.com (Z.Z.); 15139319796@163.com (Y.W.); zhoubiyan07@163.com (B.Z.); ljt1903010101@163.com (J.L.); 2 Guangxi Guigang Gangfeng Agriculture and Animal Husbandry Co., Ltd., Guigang 537100, China; lijianneng1516@163.com (J.L.); lifuqiu0908@163.com (F.L.); 3 Guangxi Key Laboratory of Animal Breeding, Disease Control and Prevention, Nanning 530004, China

**Keywords:** chicken spur, ossification pattern, testosterone, Nandan-Yao chicken, TENM2

## Abstract

The chicken spur is a bony structure located on the tarsometatarsus and extending posteriorly. Because its length increases with age, it has become an important indicator of rearing duration and a trait of interest in high-quality chicken breeding. The developmental pattern of the chicken spur and its underlying genetic mechanisms remain poorly understood. In this study, Nandan-Yao chickens were used to investigate spur growth and development. Phenotypic measurements and X-ray imaging were performed to characterize spur growth, and RNA sequencing was used to identify candidate genes associated with spur length. Candidate genes were further evaluated by tissue expression profiling and RNA interference. The results showed that spur length was not significantly associated with body weight, body size, or laying traits (*p* > 0.05). In roosters, sperm motility and ln(T/E2) were significantly higher in the long spur group than in the short spur group (*p* < 0.05). X-ray imaging and gene expression analysis indicated that the chicken spur is a bony structure that develops through an endochondral ossification pattern, with the ossification center appearing at approximately 15 weeks of age in Nandan-Yao chickens. *TENM2* was identified as a key candidate gene associated with spur development, and functional analysis suggested that it may be involved in this process through the BMP signaling pathway. These findings provide a basis for understanding the developmental pattern and genetic mechanisms of chicken spur formation and support the potential breeding value of spur traits.

## Simple Summary

The chicken spur is a bony structure that grows from the lower leg and increases in length with age. It is widely used to estimate rearing time and is an important trait in poultry breeding. However, its growth pattern and underlying biological mechanisms remain unclear. In this study, we characterized spur growth, examined its structure using imaging methods, and analyzed gene expression in spur tissues. The results showed that spur length had no significant association with body weight, body size, or egg production. Spur growth was influenced by hormones, and roosters with longer spurs showed higher sperm motility. Structural analysis confirmed that the spur is bone tissue that forms through endochondral ossification, and that a key developmental stage occurs at around 15 weeks of age. We also identified *TENM2* as a key gene that may regulate spur development through the BMP signaling pathway. These findings provide useful insights for poultry breeding and management.

## 1. Introduction

Chicken is an important source of high-quality animal protein for humans and contributes substantially to global meat consumption. In addition to production traits, several external morphological characteristics are also of breeding interest in indigenous and high-quality chicken breeds. Among these, the spur is an important phenotypic trait whose length generally increases with age, making it a potential indicator of rearing duration. Accordingly, spur length has gradually been incorporated into breeding objectives. Previous studies have shown that spur length is a moderately to highly heritable quantitative trait in chickens and may be associated with growth-related traits and female reproductive performance, suggesting that this trait has both biological significance and potential breeding value [1]. However, its relationships with production traits and the genetic mechanisms underlying spur development remain poorly understood.

The chicken spur is a bony outgrowth located on the tarsometatarsus and extending posteriorly [2]. Its development begins during embryogenesis and is closely associated with tissue growth and ossification. Although several ossification models have been proposed [3], including endochondral ossification [4], intramembranous osteogenesis [5], chondrocyte to osteogenic precursor transition [6], dedifferentiation followed by redifferentiation [7], and direct transdifferentiation [8], the ossification pattern of the chicken spur remains unclear. Among these, endochondral ossification and intramembranous ossification are the two principal modes of skeletal development [9,10,11]. Clarifying which mode is involved in spur formation is therefore important for understanding its developmental basis. Recent genetic studies have identified several candidate genes associated with spur length in chickens, but the developmental pattern of the chicken spur and the molecular mechanisms underlying its formation remain largely unclear [1].

Spur development is affected by multiple factors, including genetic background, hormonal regulation, and environmental conditions [12,13]. Previous studies have shown that gonadal and thyroid hormones play essential roles in spur growth [14], particularly in roosters, in which spur development is closely associated with secondary sexual characteristics [15]. These findings indicate that spur development is regulated by complex molecular networks and may be genetically associated with other traits, such as body size, body weight, and reproductive performance.

In this study, Nandan-Yao chickens were used to investigate spur growth patterns and the molecular basis of spur development. Phenotypic measurements and X-ray analysis were conducted to characterize spur growth, and RNA-seq was used to screen candidate genes associated with spur length. Key candidate genes were further evaluated by tissue expression profiling and preliminary functional validation. This study provides a basis for understanding the developmental pattern and genetic architecture of chicken spur formation.

## 2. Materials and Methods

### 2.1. Animal and Sample Collection

A total of 500 Nandan-Yao chickens (250 males and 250 females) were included in this study. All chickens were raised under standard feeding conditions at Guangxi Guigang Gangfeng Agriculture and Animal Husbandry Co., Ltd. (Guigang, China). Body weight and body size traits were recorded at 1 day (1D), 6 weeks (6W), 10 weeks (10W), 13 weeks (13W), 15 weeks (15W), 17 weeks (17W), and 20 weeks (20W). At 20 weeks of age, roosters were assigned to the long spur (LS) and short spur (SS) groups according to spur length, with 16 chickens per group. At 23 weeks of age, 12 roosters from each group were selected for semen quality evaluation. For X-ray analysis, four roosters were sampled at each of six time points (14W, 15W, 20W, 21W, 24W, and 53W) to investigate spur bone development.

To evaluate the relationship between spur length and egg production performance, an independent cohort of 327 hens at 40 weeks of age was additionally selected. Spur length and egg production data were collected, and the chickens were grouped according to spur length. For molecular analyses, blood and spur samples were collected from four chickens in each of the LS and SS groups at 20 weeks of age. Blood samples were used for ELISA, whereas spur tissues were used for gene expression analysis and RNA sequencing. In addition, spur samples from 1-day-old roosters were collected for RNA sequencing. Three pooled samples were prepared, each containing tissues from 10 chickens.

### 2.2. Test of Semen Quality and Serum Hormone Concentrations

Semen quality in roosters was evaluated by microscopic examination. Briefly, 10 μL of semen was collected using a pipette, mounted on a glass slide, and observed under a microscope to assess sperm motility. Blood samples were centrifuged to obtain serum, and serum estradiol (E2) and testosterone (T) concentrations were determined using chicken ELISA kits (Shanghai Enzyme Biotechnology Co., Ltd., Shanghai, China).

### 2.3. RNA-Seq Sequencing Data Analysis

Total RNA was extracted from spur tissues using TRIzol reagent (Life Technologies, Carlsbad, CA, USA) according to the manufacturer’s instructions. RNA quality and concentration were evaluated using a Q5000 UV–Vis spectrophotometer (Quawell, San Jose, CA, USA) and agarose gel electrophoresis. RNA sequencing was conducted by Novogene Bioinformatics Technology Co., Ltd. (Beijing, China) according to the standard protocol.

Raw reads were filtered using Trimmomatic (v0.39) to generate clean reads [15]. Read quality, including sequencing error rate, GC content distribution, and Q20/Q30 scores, was assessed using FastQC (v0.11.09) [16]. The version of the chicken reference genome is GRCg6a. The reference genome and annotation files were downloaded from the genome database (http://ftp.ensembl.org/pub/release-95/, accessed on 12 May 2026) accessed on 12 May 2026. Clean reads were aligned to the reference genome using HISAT2 (v2.2.1), and SAM files were converted to BAM files using SAMtools [17]. Gene expression counts were calculated using HTSeq-count (v0.13.5) [18]. Differential expression analysis was performed using the DESeq2 package (v1.38.3) in R, and genes with *p* < 0.05 and |log2FoldChange| > 1.0 were defined as differentially expressed genes (DEGs) [19]. Gene Ontology (GO) and Kyoto Encyclopedia of Genes and Genomes (KEGG) enrichment analyses were performed using the clusterProfiler package (v4.6.2) [20].

### 2.4. cDNA Synthesis and Real-Time Fluorescent Quantitative PCR (QRT-PCR)

HiScript^®^ III RT SuperMix for qPCR (+gDNA wiper) (Vazyme, Nanjing, China) was utilized for reverse transcription to synthesize cDNA. QRT-PCR was performed using Universal SYBR qPCR Master Mix (Vazyme, Nanjing, China). The amplification procedure consisted of an initial denaturation at 95 °C for 30 s, followed by 40 cycles of denaturation at 95 °C for 5 s, and annealing and extension at 60 °C for 30 s. β-actin was used as the internal reference gene, and all samples were analyzed in triplicate. Relative gene expression levels were calculated using the 2^−ΔΔCT^ method. Primer sequences are listed in Table S1.

### 2.5. RNA Interference and Cell Transfection

siRNA sequences targeting the coding sequence of the chicken *TENM2* gene were designed using the online siRNA Selection Program and synthesized by Sangon Biotech (Shanghai, China). The siRNA sequence information is listed in Table S2. siRNA3-*TENM2*, which showed the highest knockdown efficiency, was selected for subsequent experiments.

Chondrocytes were isolated from the tibial tissues of E15 chicken embryos. Tibial growth plate tissues were collected under sterile conditions, sequentially digested with trypsin and 0.2% type IV collagenase, and the isolated cells were filtered, centrifuged, and resuspended in DMEM supplemented with 10% fetal bovine serum for culture. For chondrocyte identification, third-passage cells were collected, and the relative expression levels of chondrocyte marker genes, including *ACAN*, *SOX9*, *COL2A1*, and *COL10A1*, were examined by QRT-PCR using a chicken preadipocyte cell line as the control. Cells were seeded into six-well plates and transfected with siRNA using Lipofectamine^®^ RNAiMAX (Invitrogen, Carlsbad, CA, USA) at approximately 60% confluence. After 48 h of transfection, the cells were observed, photographed, and harvested for subsequent analyses.

### 2.6. Statistical Analysis

All data are presented as the mean ± SEM. Differences between the two groups were analyzed using a two-tailed Student’s *t*-test, and *p* < 0.05 was considered statistically significant; * *p* < 0.05, ** *p* < 0.01. GraphPad Prism 8 (GraphPad Software, San Diego, CA, USA) was used for statistical analysis and data visualization. All data are presented as the results of three technical and biological replicates.

## 3. Results

### 3.1. Effects of Chicken Spur Length on Production Performance and Hormone Levels

**Figure 1:**
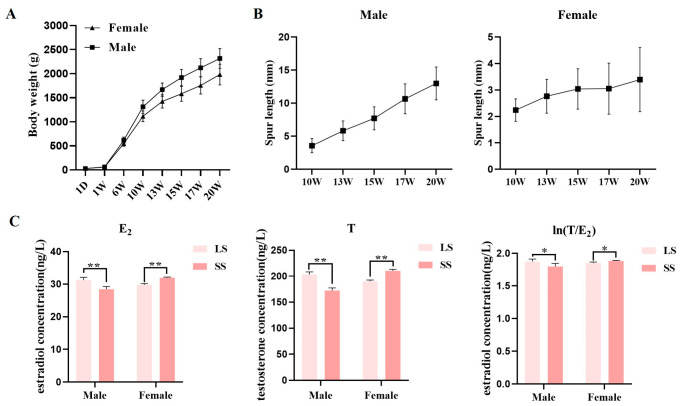
Effects of Nandan-Yao chicken spur on production performance and hormone levels. (**A**,**B**) Lines of body weight and spur length between female and male Nandan-Yao chickens in different periods; (**C**) The results of estradiol and testosterone in Nandan-Yao chickens. * *p* < 0.05, ** *p* < 0.01.

Phenotypic measurements showed that body weight in Nandan-Yao chickens increased progressively with age (Figure 1A, Table S3). Significant sex differences were observed in spur development (Figure 1B). Hens showed delayed spur growth, whereas roosters exhibited a marked and continuous increase in spur length from 10 weeks of age onward. Because spur growth in hens was limited, correlation analyses with growth traits were performed only in roosters at 15, 17, and 20 weeks of age. As shown in Table 1, spur length in 15-week-old roosters was highly significantly positively correlated with comb thickness (*p* < 0.01), and significantly negatively correlated with shank length and shank circumference (*p* < 0.05). In 17-week-old roosters, spur length was significantly negatively correlated with body length (*p* < 0.01), whereas no significant correlations were found for the other traits. In 20-week-old roosters, spur length was significantly negatively correlated with shank length (*p* < 0.01).

In hens, spur length was not significantly correlated with laying performance traits, including total egg number, qualified egg number, and qualified egg rate (*p* > 0.05) (Table S4), and no significant differences in laying performance were observed between the LS and SS groups (Table S5). In roosters, sperm density did not differ significantly between groups, whereas sperm motility was significantly higher in the LS group than in the SS group (*p* < 0.05) (Table 2). In addition, serum E2 concentration, T concentration, and the ln(T/E2) ratio were significantly higher in the LS group than in the SS group in roosters, whereas the opposite pattern was observed in hens (*p* < 0.05) (Figure 1C, Table S6).

### 3.2. The Ossification Process of Spur

**Figure 2:**
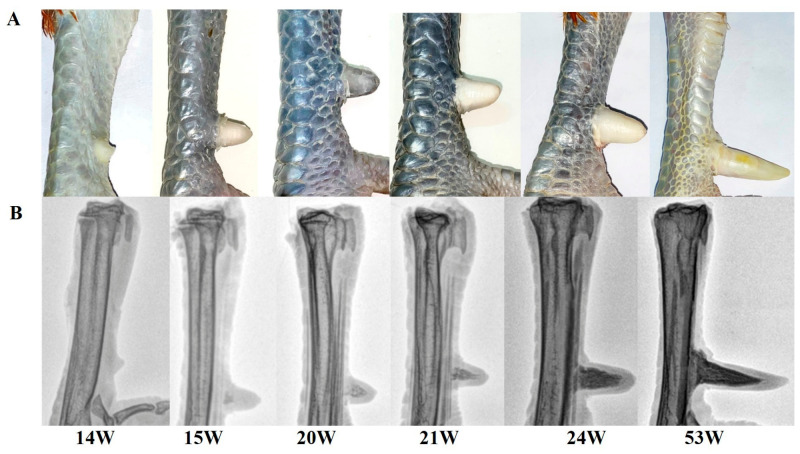
The X-ray photos of rooster spur in different times. (**A**) white light; (**B**) X-Ray.

The ossification process of the spur was evaluated by DR X-ray imaging at different developmental stages. As shown in Figure 2, spur ossification progressively increased during development. At the early developmental stage, the spur was short and showed no obvious internal ossification. By 15 weeks of age, a distinct ossification center had appeared within the spur, suggesting that this stage represented a key period in early spur ossification. At approximately 20 weeks, the spur had elongated further, and the ossification center had enlarged and extended toward the tarsometatarsus, indicating progressive ossification. By 21 weeks, the ossification center had begun to contact the tarsometatarsus, and by 24 weeks, this contact had become closer as the spur continued to extend. Radiographic density increased progressively throughout development, indicating enhanced calcification and mineralization. By 53 weeks, the spur exhibited a high degree of mineralization and bone density, with complete ossification and firm fusion to the tarsometatarsus.

### 3.3. Expression of Key Ossification Genes in the Spur and Other Bone Tissues

**Figure 3:**
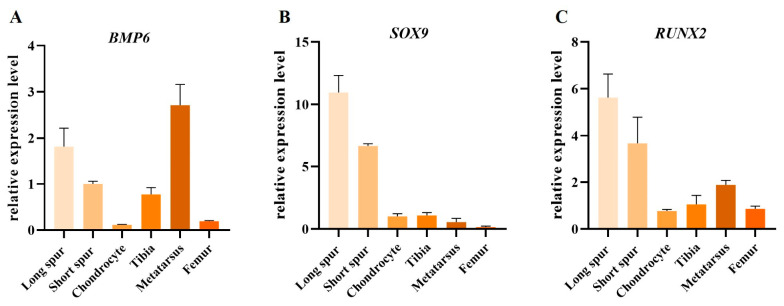
Expression of key ossification genes in the spur and other bone tissues. (**A**–**C**) The expression levels of key ossification genes *BMP6*, *SOX9*, and *RUNX2*.

To compare the molecular characteristics of the spur with those of other bone tissues, the expression levels of *BMP6*, *RUNX2*, and *SOX9* were analyzed by QRT-PCR in long spur, short spur, chondrocytes, metatarsus, and femur tissues. The expression levels of all three genes were significantly higher in the long spur than in the short spur (*p* < 0.01) (Figure 3A–C). *BMP6* expression was highest in the metatarsus and lowest in chondrocytes, whereas *RUNX2* and *SOX9* showed the highest expression in the long spur. The lowest expression of *RUNX2* was detected in chondrocytes, while *SOX9* was lowest in the femur. These findings suggest that *BMP6*, *RUNX2*, and *SOX9* are involved in spur growth and ossification, and that *SOX9* may have a particularly important role in spur development.

### 3.4. RNA-Seq, GO, and KEGG Analysis

**Figure 4:**
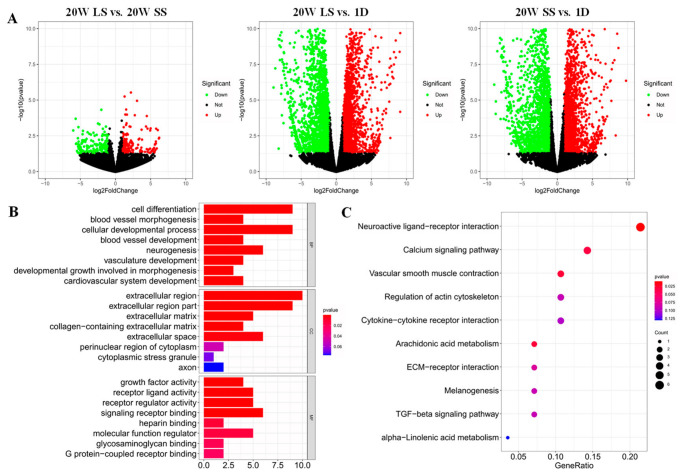
Differential expression genes (DEGs) analysis and GO, KEGG enrichment analysis. (**A**) Volcano plot of DEGs in 20W LS vs. 20W SS, 20W LS vs. 1D, 20W SS vs. 1D. 20W LS: 20-week long spur group; 20W SS: 20-week short spur group; 1D: 1-day-old spur group. (**B**) GO functional classification map of intersection genes of each comparison group DEGs. (**C**) Bubble plots of the KEGG pathway for the intersection genes of each comparison group DEGs.

A total of 11 samples were subjected to RNA-seq, and the sequencing results and quality control parameters are summarized in Table S7. Five DEGs were selected for validation by QRT-PCR, and the results were consistent with the RNA-seq data, supporting the reliability of the sequencing results (Figure S1). Differential expression analysis identified 333, 4594, and 4180 DEGs in the comparisons of 20-week-old long spur group (20W LS) vs. 20-week-old short spur group (20W SS), 20W LS vs. 1-day-old spur group (1D), and 20W SS vs. 1D, respectively (Figure 4A). Intersection analysis of the three DEG sets identified 80 common genes, which may be involved in both spur development and spur length variation (Figure S2).

GO enrichment analysis showed that DEGs in the 20W LS vs. 20W SS comparison were mainly associated with cell differentiation, cellular developmental processes, negative regulation of canonical WNT signaling, and regulation of programmed cell death. In the 20W LS vs. 1D comparison, enriched GO terms were mainly related to anatomical structure morphogenesis, animal organ development, skeletal system development, and embryonic appendage morphogenesis. In the 20W SS vs. 1D comparison, enriched functions were primarily associated with skeletal system development, embryonic appendage morphogenesis, cell differentiation, and embryonic limb morphogenesis (Figure S3). The 80 common DEGs were mainly enriched in cell differentiation, blood vessel morphogenesis, cellular developmental processes, growth-related morphogenesis, and cardiovascular system development (Figure 4B, Table S8).

KEGG analysis showed that DEGs in the 20W LS vs. 20W SS comparison were mainly enriched in the MAPK signaling pathway, cytokine–cytokine receptor interaction, cell adhesion molecules, and the WNT signaling pathway. In the 20W LS vs. 1D comparison, the DEGs were mainly enriched in cell adhesion molecules, glycerophospholipid metabolism, and sphingolipid metabolism. In the 20W SS vs. 1D comparison, the DEGs were mainly enriched in the MAPK signaling pathway, cell adhesion molecules, glycerolipid metabolism, and the PPAR signaling pathway. The 80 common DEGs were mainly enriched in neuroactive ligand–receptor interaction, vascular smooth muscle contraction, and calcium signaling pathways (Figure 4C, Table S9).

### 3.5. Identification of Important Candidate Genes Related to Chicken Spur Development

**Figure 5:**
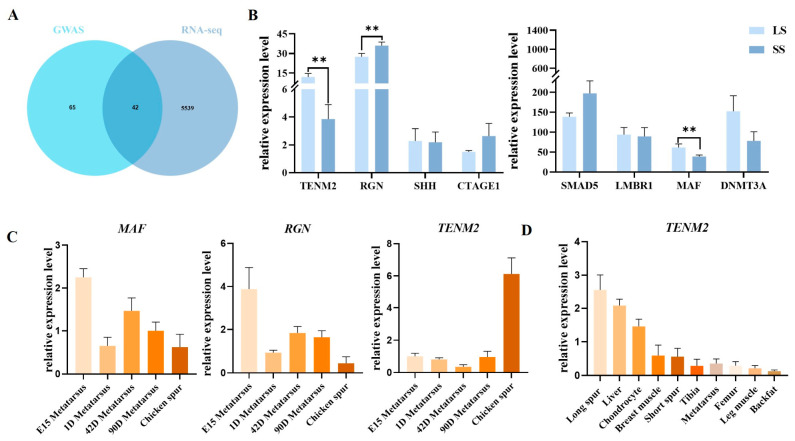
Screening of important candidate genes related to chicken spur development. (**A**) The transcriptome DEGs overlapped with the GWAS result gene. (**B**) QRT-PCR detection results of the expression of DEGs in RNA-Seq. (**C**) QRT-PCR detection results of *MAF*, *RGN* and *TENM2* in spur and metatarsus. (**D**) Tissue expression profile of *TENM2* genes. LS: long spur group, SS: short spur group. ** *p* < 0.01.

Combined with our previous genome-wide association study (GWAS), the DEGs identified from the transcriptome analysis were intersected with the candidate genes obtained from the GWAS results (Figure 5A). GO functional enrichment analysis was then performed for the overlapping genes, and the results are shown in Table 3. These genes were mainly enriched in biological processes related to limb morphogenesis, connective tissue development, cartilage development, calcium signaling, and appendage morphogenesis. Based on the integrated analysis, eight candidate genes, namely *MAF*, *TENM2*, *RGN*, *SHH*, *LMBR1*, *CTAGE1*, *SMAD5*, and *DNMT3A*, were identified as potentially associated with chicken spur development.

To further identify key genes involved in spur growth, QRT-PCR was performed to examine the expression of these candidate genes in spur and metatarsal tissues. The results showed that the expression levels of *TENM2*, *RGN*, and *MAF* in the LS were significantly higher than in the SS (*p* < 0.01) (Figure 5B). *TENM2* exhibited significantly high expression in the chicken spur (*p* < 0.01), while RGN and MAF demonstrated the highest expression levels in the 15D embryonic metatarsal bones (*p* < 0.01) (Figure 5C). In addition, *TENM2* showed the highest expression in the long spur (Figure 5D). Based on these findings, *TENM2* was considered the key candidate gene associated with chicken spur length and was selected for further investigation.

### 3.6. TENM2 Function in Chondrocytes

**Figure 6:**
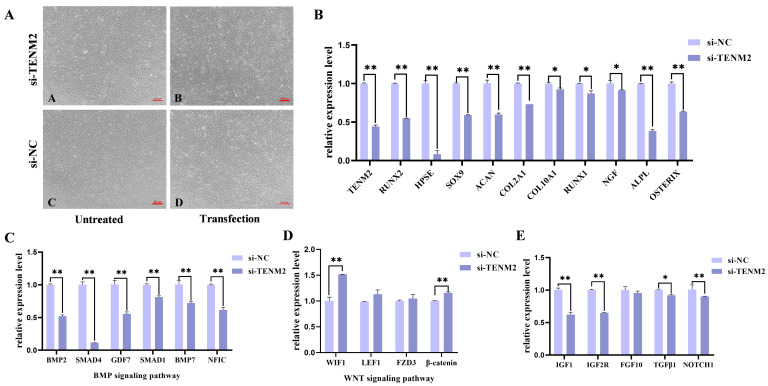
Effect of *TENM2* interference in chicken chondrocytes. (**A**) Images of chicken chondrocytes before and after 48 h of transfection (scale bars: 200 μm), QRT-PCR to detect the expression of *TENM2* gene; (**B**) QRT-PCR to detect the expression of marker genes after *TENM2* interference; (**C**,**D**) QRT-PCR to detect the expression of BMP and WNT signaling pathway genes after *TENM2* interference; (**E**) QRT-PCR to detect the expression of endochondral ossification genes after *TENM2* interference; * *p* < 0.05, ** *p* < 0.01.

Before functional analysis, the isolated cells were identified by QRT-PCR detection of chondrocyte marker genes. The relative expression levels of *ACAN*, *SOX9*, *COL2A1*, and *COL10A1* were all significantly higher in the isolated cells than in the chicken preadipocyte cell line (*p* < 0.05), confirming the successful isolation of chondrocytes (Figure S4). To investigate the role of *TENM2* in chicken chondrocytes, si-*TENM2* and si-NC were transfected into chondrocytes. After 48 h of transfection (Figure 6A,B), TENM2 expression was significantly reduced in the si-*TENM2* group compared with the control group (*p* < 0.01). The expression levels of *RUNX2* and *HPSE*, which are associated with chondrocyte proliferation, were significantly decreased (*p* < 0.01). In addition, the expression levels of differentiation-related genes (*SOX9*, *ACAN*, *COL2A1*, *COL10A1*, and *RUNX1*) and apoptosis-related genes (*NGF* and *ALPL*) were significantly reduced (*p* < 0.05), while the hypertrophic chondrocyte marker *OSTERIX* was also significantly downregulated (*p* < 0.01). These findings suggest that *TENM2* interference inhibits chondrocyte proliferation, differentiation, hypertrophy, and apoptosis.

The effects of *TENM2* interference on bone development-related signaling pathways were further examined (Figure 6C,D). Compared to the control group, the expression levels of WNT pathway genes exhibited an increase following *TENM2* interference, and the relative expression of WIF1 and β-catenin genes was significantly increased (*p* < 0.05). In contrast, the expression levels of BMP pathway genes were significantly decreased (*p* < 0.01). These results suggest that *TENM2* primarily influences chondrocyte development through the BMP signaling pathway, thereby impacting the development of the chicken spur.

The relative expression levels of genes involved in bone growth and development were assessed (Figure 6E). Compared to the control group, interference with *TENM2* significantly decreased the expression levels of *IGF1*, *IGF2R*, *TGFβ1*, and *NOTCH1* (*p* < 0.05). These results further confirm that *TENM2* plays a crucial role in chondrocyte development, identifying it as a key gene influencing the development of the chicken spur.

## 4. Discussion

At present, relatively few studies have explored the relationships between chicken spur length and other traits. In this study, the associations between spur length and growth traits in roosters differed across developmental stages, and no significant correlation with body weight was detected. These findings suggest that spur length is not stably associated with general growth performance, but may be related to specific morphological traits during particular developmental periods. With respect to reproductive traits, sperm density did not differ significantly between the LS and SS groups, whereas sperm motility was significantly higher in the LS group, consistent with previous findings [21]. This result suggests that longer spurs in roosters may be associated with certain aspects of reproductive fitness. In contrast, no significant relationship was observed between spur length and laying performance in hens, indicating that spur length may have limited relevance to female reproductive performance in Nandan-Yao chickens. A previous study in White Leghorn hens reported a negative phenotypic correlation between spur length and egg production [22], which differs from the present findings. A study in Rhode Island Red chickens showed that spur length had moderate to high heritability and was positively correlated with body weight, shank length, age at first egg, and body weight at first egg [1]. These discrepancies among studies may reflect differences in genetic background, selective breeding history, trait definition, and possible genotype–environment interactions across breeds. This inconsistency may be due to differences in breed, genetic background, rearing environment, or management conditions. In addition, sex hormones are known to play critical roles in skeletal growth and development. In the present study, serum E2 and T levels were significantly higher in the LS group than in the SS group in roosters, whereas the opposite pattern was observed in hens, suggesting sex-specific hormonal regulation of spur growth. Taken together, these findings indicate that selection for spur length is unlikely to adversely affect the measured production traits and may even be beneficial for sperm motility in roosters [23]. Therefore, spur length may have potential value as an auxiliary trait in specific breeding contexts, although this possibility still requires further validation.

In this study, X-ray imaging was used to monitor changes in spur ossification throughout its growth and development. Because cartilage tissue cannot be clearly visualized by X-ray imaging, no obvious ossified structure was detected within the spur before 14 weeks of age, suggesting that the spur remained at an early developmental stage during this period. At approximately 15 weeks of age, a small ossification focus first appeared in the inner region of the spur, which subsequently developed into an ossification center. As development progressed, the ossification center gradually extended toward and eventually fused with the tarsometatarsus. This transition became evident at approximately 20–21 weeks of age in Nandan-Yao chickens, and initial fusion was observed by 24 weeks. By 53 weeks of age, the spur showed complete ossification and firm fusion with the tarsometatarsus, forming the bony base of the spur. These findings were generally consistent with previous reports [24], although differences in developmental timing were observed, which may be related to breed differences and long-term artificial selection [25].

X-ray analyses revealed that the spur belongs to bone tissue, and its developmental pattern is consistent with endochondral ossification. To further evaluate this possibility, the expression of key ossification-related genes was examined. The results showed that *SOX9* and *RUNX2* were expressed in the spur, and *SOX9* expression was significantly higher in the LS group than in the SS group. As *SOX9* is a key regulator of chondrogenesis and *RUNX2* is essential for osteogenic differentiation and chondrocyte maturation [26], the differential expression of these genes suggests that differences in cartilage development and ossification progression may contribute to variation in spur length. Previous studies have also shown that regulation of chondrocyte hypertrophy by the WNT signaling pathway is closely associated with *SOX9* and *RUNX2* expression [27]. Taken together, these findings support the view that the chicken spur is a bony tissue that develops through an endochondral ossification process. Although other ossification mechanisms have been proposed in previous studies, the present X-ray observations, together with the expression patterns of cartilage and bone-related markers, more strongly support an endochondral ossification pattern in chicken spur development. However, its precise developmental and regulatory mechanisms remain to be further elucidated.

The growth and development of the chicken spur is a complex biological process regulated by multiple genes and signaling pathways. Recent genomic studies have shown that spur length is a heritable trait and have identified multiple candidate genes associated with this phenotype through Pool-GWAS and selection signature analyses, supporting the view that spur development has a complex genetic basis [1]. In the present study, RNA-seq analysis of spur tissues from 1-day-old and 20-week-old Nandan-Yao roosters identified candidate genes associated with spur development. Combined with the observed ossification pattern, GO and KEGG enrichment analyses suggested that variation in spur length may be closely related to cartilage development and signaling pathways such as calcium signaling and WNT signaling. By integrating the RNA-seq data with previous GWAS results, eight candidate genes associated with spur development were identified, among which *TENM2* was further recognized as a key candidate gene associated with spur length. Previous studies have suggested that *TENM2* may respond to *FGFR2*-mediated signaling and exhibit an expression pattern similar to that of *FGF8* [28]. FGF8 promotes cell differentiation and enhances osteogenic activity [29], *TENM2* may influence chicken spur growth and development through related regulatory mechanisms. However, the precise molecular mechanism requires further investigation.

During bone growth and development, chondrocyte proliferation, differentiation, hypertrophy, and osteoblast differentiation are regulated by multiple transcription factors and signaling pathways [30]. *RUNX2* regulates bone formation and remodeling by promoting vascular infiltration and chondrocyte maturation [31]. When *HPSE* gene expression is inhibited, osteogenic differentiation is enhanced, while cartilage formation is suppressed [32]. *SOX9*, as a transcription factor, marks the formation of bone progenitor cells and directs their differentiation into chondrocytes [33]. *COL10A1*, *COL2A1*, and *ACAN* are important extracellular matrix-associated genes in growth plate chondrocytes, and *ACAN* and *COL2A1* are widely recognized as markers of chondrocyte differentiation [34]. *ALPL* encodes alkaline phosphatase, and phosphates promote chondrocyte mineralization and apoptosis through the MAPK signaling pathway [35]. *NGF* induces chondrocyte apoptosis by regulating *ASIC1a* expression [36]. During endochondral ossification, the Hedgehog, BMP, and WNT signaling pathways interact to regulate skeletal development [37,38]. In this study, *TENM2* interference significantly reduced the expression of genes associated with chondrocyte proliferation, differentiation, hypertrophy, and apoptosis-related processes. Moreover, opposite expression changes were observed in WNT and BMP-related genes after *TENM2* interference, suggesting potential crosstalk between these pathways. Collectively, these findings indicate that *TENM2* may influence chicken spur development by modulating chondrocyte developmental processes, at least in part through the BMP signaling pathway.

## 5. Conclusions

In conclusion, this study demonstrated that the chicken spur is a bony structure that develops through endochondral ossification and identified the key developmental stage for ossification center formation in Nandan-Yao chickens. Significant sex differences were observed in spur growth, and roosters in the long spur group showed a higher ln(T/E2) ratio and greater sperm motility than those in the short spur group. Spur length was not adversely associated with growth performance. Furthermore, *TENM2* was identified as a key candidate gene associated with spur length, and functional analyses suggested that it may regulate chondrocyte development and spur formation primarily through the BMP signaling pathway. These findings mainly improve the understanding of the developmental pattern and molecular basis of chicken spur formation.

## Figures and Tables

**Table 1:** Correlation analysis between spur length and body measurement traits of male Nandan-Yao chickens in different periods.

Weeks	Index	BW	BL	KL	HW	CH	CL	CT	SL	SC
15W	Spur length	−0.066	−0.069	0.072	−0.055	0.15	0.075	0.223 **	−0.168 *	−0.198 *
17W		−0.029	−0.286 **	0.037	0.091	0.074	0.071	0.089	0.064	0.019
20W		−0.003	0.016	−0.159	−0.080	0.153	−0.024	0.078	−0.195 *	0.021

BW: Body weight, BL: Body length, KL: Keel length, SC: Shank circumference, HW: Hip width, CH: Comb height, CL: Comb length, CT: Comb thickness, SL: Shank length, SC: Shank circumference. * *p* < 0.05, ** *p* < 0.01.

**Table 2:** Comparison of semen quality of long spur and short spur in male Nandan-Yao chickens.

	Long Spur Group (*n* = 12)	Short Spur Group (*n* = 12)
Weeks	23 W	23 W
Spur length (mm)	19.2 ± 0.58 ^A^	9.84 ± 1.18 ^B^
Sperm density/(billion·mL^−1^)	9.61 ± 2.45	8.97 ± 2.28
Sperm motility (%)	77.47 ± 13.58 ^A^	63.14 ± 13.14 ^B^

Uppercase letters indicate *p* < 0.01.

**Table 3:** GO function-enriched differential genes.

ID	Description	*p* Value	Count	Gene ID
GO:0030326	embryonic limb morphogenesis	0.006566	3	*LMBR1*/*SHH*/*TBX5*
GO:0035113	embryonic appendage morphogenesis	0.007458	3	*LMBR1*/*SHH*/*TBX5*
GO:0035108	limb morphogenesis	0.011722	3	*LMBR1*/*SHH*/*TBX5*
GO:0035107	appendage morphogenesis	0.012967	3	*LMBR1*/*SHH*/*TBX5*
GO:0042733	embryonic digit morphogenesis	0.013	2	*LMBR1*/*SHH*
GO:0051216	cartilage development	0.017864	4	*SHH*/*CTAGE1*/*MAF*/*SMAD5*
GO:0061448	connective tissue development	0.018943	4	*SHH*/*CTAGE1*/*MAF*/*SMAD5*
GO:0019722	calcium-mediated signaling	0.025125	2	*RGN*/*TENM2*
GO:0060173	limb development	0.029516	3	*LMBR1*/*SHH*/*TBX5*
GO:0071407	cellular response to organic cyclic compound	0.029516	3	*UFM1*/*GABRG2*/*SMAD5*

## Data Availability

The original contributions presented in this study are included in the article/Supplementary Material. Further inquiries can be directed to the corresponding author.

## Supplementary Materials

The following supporting information can be downloaded at: https://www.mdpi.com/article/10.3390/ani16111577/s1. Figure S1: The accuracy of RNA-seq was verified by QRT-PCR; Figure S2: Venn diagram of DEGs in each comparison group; Figure S3: GO and KEGG pathway enrichment analyses of DEGs in 20W LS vs. 20W SS, 20W LS vs. 1D and 20W LS vs. 1D; Figure S4: The relative expression level of mRNA for the chondrocyte marker genes. Table S1: Primer information for QRT-PCR; Table S2: TENM2 siRNA sequence information; Table S3: The Weight of Nandan-Yao chickens in different periods; Table S4: Correlation analysis between spur length and laying performance of female Nandan-Yao chickens; Table S5: Comparison of laying performance of long spur and short spur in female Nandan-Yao chickens; Table S6: The measurement results of estradiol and testosterone; Table S7: Quality analysis of investigated samples; Table S8: Top 10 most significantly enriched Gene Ontology (GO) terms for DEGs; Table S9: significantly enriched KEGG pathways for DEGs.
