# Supplementary material for: Exploring Growth Pattern and Candidate Genes for Chicken Spur
Source: Animals (Basel). 2026 May 22;16(11):1577. doi: 10.3390/ani16111577 (PMC13255967; doi:10.3390/ani16111577)
Supplement: Supplementary file 1 [file animals-16-01577-s001.zip › animals-4293732-Supplementary Figures.pdf]

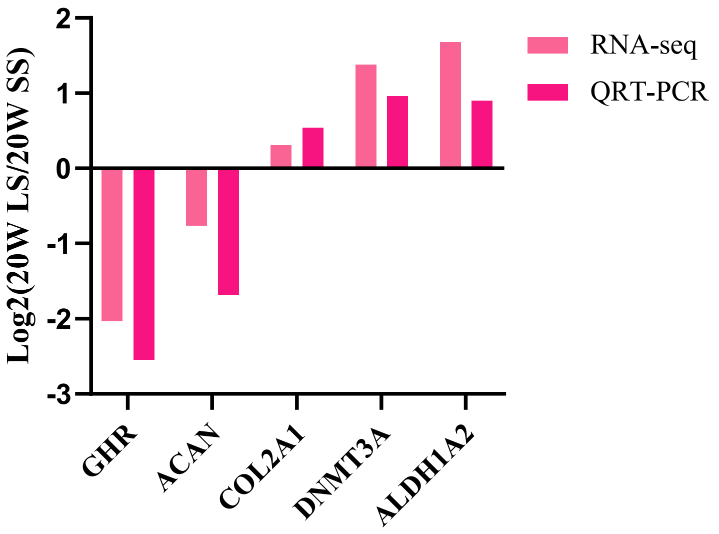

Figure S1. The accuracy of RNA-seq was verified by QRT-PCR.

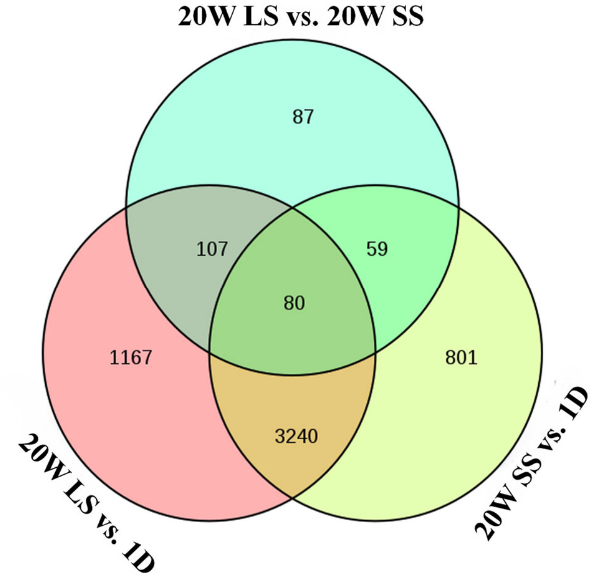

Figure S2. Venn diagram of DEGs in each comparison group.

A

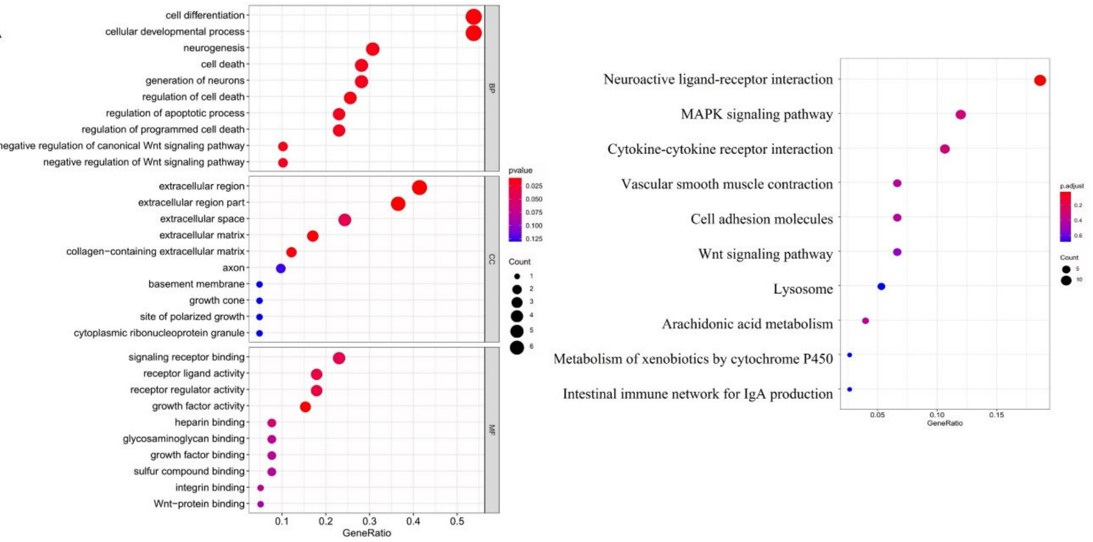
cell differentiation
cellular developmental process
neurogenesis
cell death
generation of neurons
regulation of cell death
regulation of apoptotic process
regulation of programmed cell death
negative regulation of canonical Wnt signaling pathway
negative regulation of Wnt signaling pathway
extracellular region
extracellular region part
extracellular space
extracellular matrix
collagen−containing extracellular matrix
axon
basement membrane
growth cone
site of polarized growth
cytoplasmic ribonucleoprotein granule
signaling receptor binding
receptor ligand activity
receptor regulator activity
growth factor activity
heparin binding
glycosaminoglycan binding
growth factor binding
sulfur compound binding
integrin binding
Wnt−protein binding
BP
CC
MF
pvalue
0.025
0.050
0.075
0.100
0.125
Count
1
2
3
4
5
6
0.1
0.2
0.3
0.4
0.5
GeneRatio
Neuroactive ligand-receptor interaction
MAPK signaling pathway
Cytokine-cytokine receptor interaction
Vascular smooth muscle contraction
Cell adhesion molecules
Wnt signaling pathway
Lysosome
Arachidonic acid metabolism
Metabolism of xenobiotics by cytochrome P450
Intestinal immune network for IgA production
p.adjust
Count
0.05
0.10
0.15
GeneRatio

B

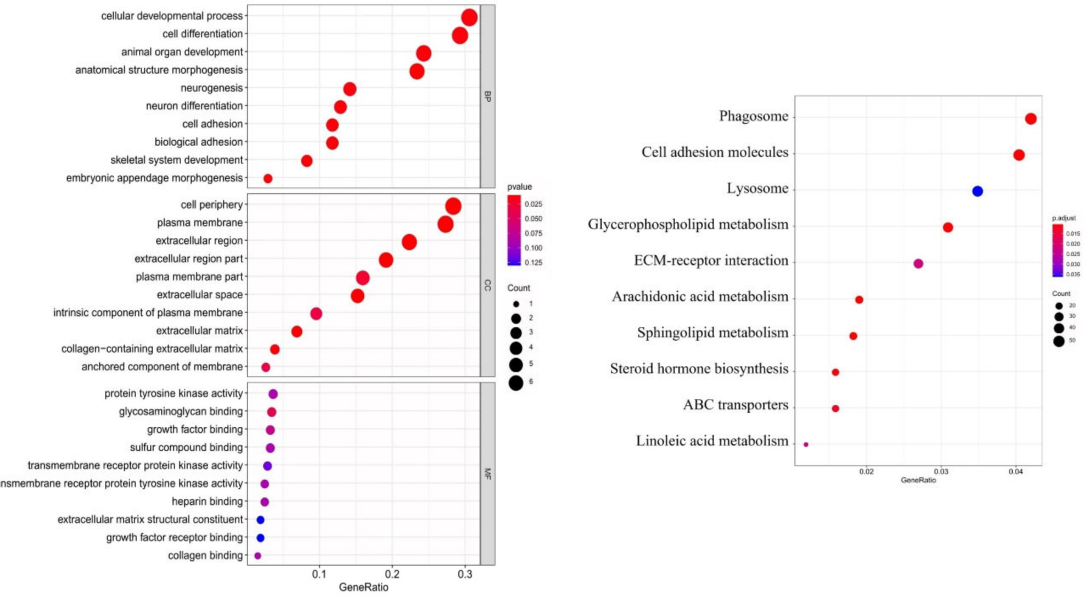
cellular developmental process
cell differentiation
animal organ development
anatomical structure morphogenesis
neurogenesis
neuron differentiation
cell adhesion
biological adhesion
skeletal system development
embryonic appendage morphogenesis
cell periphery
plasma membrane
extracellular region
extracellular region part
plasma membrane part
extracellular space
intrinsic component of plasma membrane
extracellular matrix
collagen−containing extracellular matrix
anchored component of membrane
protein tyrosine kinase activity
glycosaminoglycan binding
growth factor binding
sulfur compound binding
transmembrane receptor protein kinase activity
transmembrane receptor protein tyrosine kinase activity
heparin binding
extracellular matrix structural constituent
growth factor receptor binding
collagen binding
BP
CC
MF
pvalue
0.025
0.050
0.075
0.100
0.125
Count
1
2
3
4
5
6
0.1
0.2
0.3
GeneRatio
Phagosome
Cell adhesion molecules
Lysosome
Glycerophospholipid metabolism
ECM-receptor interaction
Arachidonic acid metabolism
Sphingolipid metabolism
Steroid hormone biosynthesis
ABC transporters
Linoleic acid metabolism
p.adjust
Count
0.02
0.03
0.04
GeneRatio

C

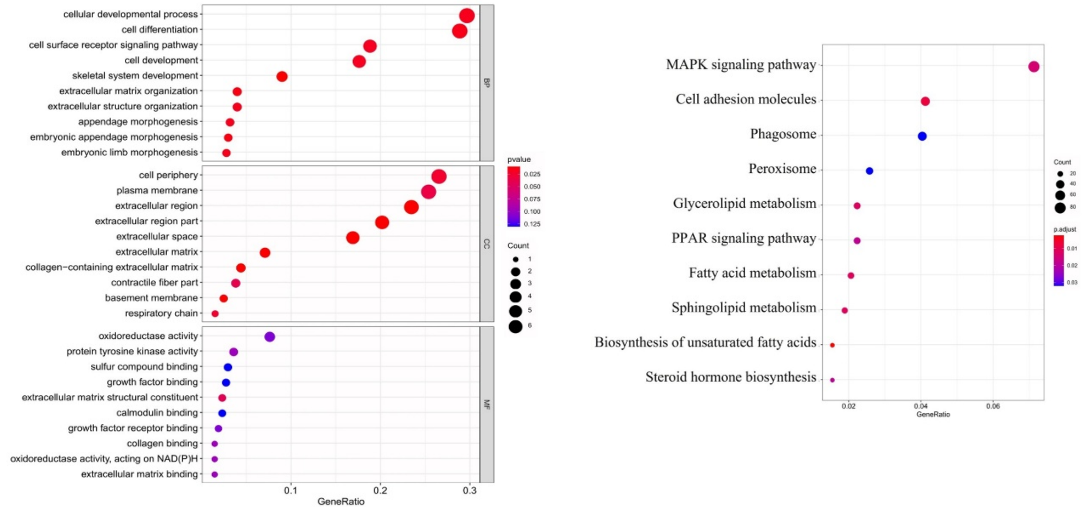
cellular developmental process
cell differentiation
cell surface receptor signaling pathway
cell development
skeletal system development
extracellular matrix organization
extracellular structure organization
appendage morphogenesis
embryonic appendage morphogenesis
embryonic limb morphogenesis
cell periphery
plasma membrane
extracellular region
extracellular region part
extracellular space
extracellular matrix
collagen−containing extracellular matrix
contractile fiber part
basement membrane
respiratory chain
oxidoreductase activity
protein tyrosine kinase activity
sulfur compound binding
growth factor binding
extracellular matrix structural constituent
calmodulin binding
growth factor receptor binding
collagen binding
oxidoreductase activity, acting on NAD(P)H
extracellular matrix binding
BP
CC
MF
pvalue
0.025
0.050
0.075
0.100
0.125
Count
1
2
3
4
5
6
0.1
0.2
0.3
GeneRatio
MAPK signaling pathway
Cell adhesion molecules
Phagosome
Peroxisome
Glycerolipid metabolism
PPAR signaling pathway
Fatty acid metabolism
Sphingolipid metabolism
Biosynthesis of unsaturated fatty acids
Steroid hormone biosynthesis
Count
p.adjust
0.02
0.04
0.06
GeneRatio

Figure S3. GO and KEGG pathway enrichment analyses of DEGs in 20W LS vs. 20W SS, 20W LS vs. 1D and 20W LS vs. 1D.

(A) 20W LS vs. 20W SS; (B) 20W LS vs. 1D; (C) 20W LS vs. 1D. 20W LS: 20-week long spur group; 20W SS: Short 20-week short spur group; 1D: 1-day-old spur group.

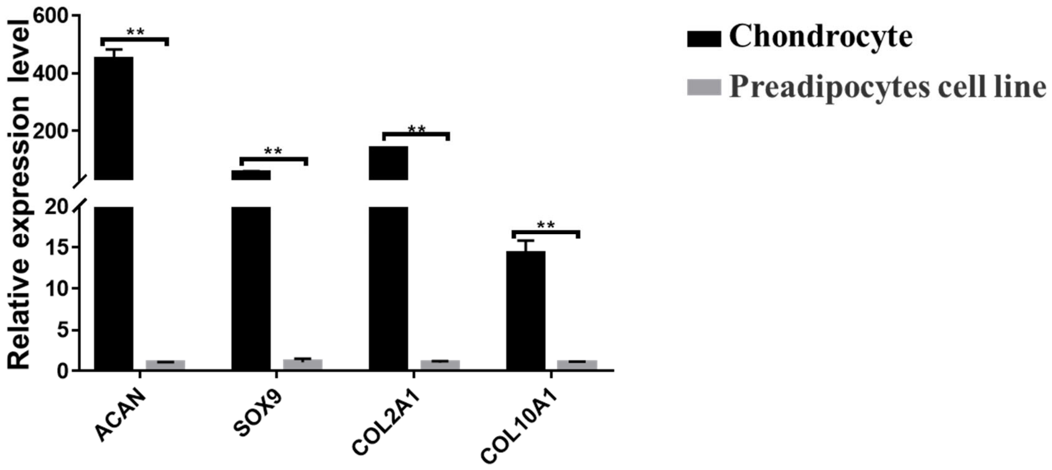

Figure S4. The relative expression level of mRNA for the chondrocyte marker genes.
